# Chotosan (*Diaoteng San*)-induced improvement of cognitive deficits in senescence-accelerated mouse (SAMP8) involves the amelioration of angiogenic/neurotrophic factors and neuroplasticity systems in the brain

**DOI:** 10.1186/1749-8546-6-33

**Published:** 2011-09-23

**Authors:** Qi Zhao, Takako Yokozawa, Koichi Tsuneyama, Ken Tanaka, Takeshi Miyata, Notoshi Shibahara, Kinzo Matsumoto

**Affiliations:** 1 Division of Medicinal Pharmacology, Institute of Natural Medicine, University of Toyama, 2630 Sugitani, Toyama 930-0194, Japan; 2 Division of Kampo Diagnostics, Institute of Natural Medicine, University of Toyama, 2630 Sugitani, Toyama 930-0194, Japan; 3 Collaboration Division, Organization for Promotion of Regional Collaboration, University of Toyama, 3190 Gofuku, Toyama 930-8555, Japan; 4 Department of Diagnostic Pathology, Graduate School of Medical and Pharmaceutical Sciences, University of Toyama, 2630 Sugitani, Toyama 930-0194, Japan; 5 Division of Pharmacognosy, Institute of Natural Medicine, University of Toyama, 2630 Sugitani, Toyama 930-0194, Japan; 6 Division of Biomedical Informatics, Institute of Natural Medicine, University of Toyama, 2630 Sugitani, Toyama 930-0194, Japan; 7 Laboratory of Presymptomatic Medical Pharmacology, Faculty of Pharmaceutical Sciences, Sojo University, 4-22-1 Ikeda, Kumamoto 860-0082, Japan

## Abstract

**Background:**

Chotosan (CTS, *Diaoteng San*), a Kampo medicine (*ie *Chinese medicine) formula, is reportedly effective in the treatment of patients with cerebral ischemic insults. This study aims to evaluate the therapeutic potential of CTS in cognitive deficits and investigates the effects and molecular mechanism(s) of CTS on learning and memory deficits and emotional abnormality in an animal aging model, namely 20-week-old senescence-accelerated prone mice (SAMP8), with and without a transient ischemic insult (T2VO).

**Methods:**

Age-matched senescence-resistant inbred strain mice (SAMR1) were used as control. SAMP8 received T2VO (T2VO-SAMP8) or sham operation (sham-SAMP8) at day 0. These SAMP8 groups were administered CTS (750 mg/kg, p.o.) or water daily for three weeks from day 3.

**Results:**

Compared with the control group, both sham-SAMP8 and T2VO-SAMP8 groups exhibited cognitive deficits in the object discrimination and water maze tests and emotional abnormality in the elevated plus maze test. T2VO significantly exacerbated spatial cognitive deficits of SAMP8 elucidated by the water maze test. CTS administration ameliorated the cognitive deficits and emotional abnormality of sham- and T2VO-SAMP8 groups. Western blotting and immunohistochemical studies revealed a marked decrease in the levels of phosphorylated forms of neuroplasticity-related proteins, N-methyl-D-aspartate receptor 1 (NMDAR1), Ca^2+^/calmodulin-dependent protein kinase II (CaMKII), cyclic AMP responsive element binding protein (CREB) and brain-derived neurotrophic factor (BDNF) in the frontal cortices of sham-SAMP8 and T2VO-SAMP8. Moreover, these animal groups showed significantly reduced levels of vasculogenesis/angiogenesis factors, vascular endothelial growth factor (VEGF), VEGF receptor type 2 (VEGFR2), platelet-derived growth factor-A (PDGF-A) and PDGF receptor α (PDGFRα). CTS treatment reversed the expression levels of these factors down-regulated in the brains of sham- and T2VO-SAMP8.

**Conclusion:**

Recovery of impaired neuroplasticity system and VEGF/PDGF systems may play a role in the ameliorative effects of CTS on cognitive dysfunction caused by aging and ischemic insult.

## Background

Chotosan (CTS, *Diaoteng San*) is a Kampo (*ie *Chinese medicine) formula consisting of ten medicinal herbs and *gypsum fibrosum*. It has long been used to treat chronic headache and hypertension, particularly in middle-aged or older patients with weak physical constitutions, chronic headache, painful tension of the shoulders and cervical muscles, vertigo, morning headache, a heavy feeling of the head, flushing, tinnitus, and insomnia [1]. In a double-blind and placebo-controlled clinical study [1], CTS showed an ameliorative effect on cognitive dysfunctions in stroke patients. CTS and tacrine (a cholinesterase inhibitor) exhibit a preventive effect on cognitive deficits in a mouse model of transient cerebral ischemia and a therapeutic effect on learning and memory impairments in a mouse model of chronic cerebral hypoperfusion [2,3]. These findings suggest that CTS may be used as an anti-dementia drug. However, since the beneficial effects of CTS have been demonstrated in young animals from eight to 15 weeks old, it is still unclear whether CTS is applicable to treat cognitive dysfunction caused by ischemic insult in aged animals.

Aging is a risk factor for a variety of diseases including deterioration of brain function [4]. One of the prominent symptoms due to aging-induced brain dysfunction is cognitive deficits such as in Alzheimer disease (AD) and cerebrovascular disease-related dementia [5]. Indeed, the incidence rates of AD and cerebrovascular dementia increase with aging [4,6]. It has been suggested that cerebrovascular diseases also play an important role in the pathogenic mechanism(s) underlying sporadic (non-genetic) AD [4,7] and that patients with AD pathology often have concomitant cerebrovascular pathology [8,9]. In fact, aging causes impaired angiogenesis that is in part attributable to a decrease in angiogenic growth factors such as VEGF [7]. Retardation of angiogenesis in the brains of aged animals is severe enough to impair synaptic plasticity, a molecular biological process important in learning and memory, and requires long-lasting increases in metabolic demand supported by the generation of new capillaries [10]. Moreover, recent evidence has shown that the VEGF and platelet-derived growth factor (PDGF), angiogenic growth factors are important not only in angiogenesis but also in neuroprotection and neurogenesis in the brain [11] and that elevation of these factors improves cognitive deficits and mental activity in aged animals [12-14]. Therefore, drugs used to treat cerebrovascular dementia or drugs with a potential to affect angiogenic factors are likely to be beneficial for cognitive dysfunctions related to aging.

The senescence-accelerated mouse (SAM) is a model of accelerated senescence established by phenotypic selection from a common genetic pool of AKR/J strain mice [15]. In particular, SAMP8 is one of the strains that exhibit early development of a variety of aging-related symptoms such as impaired immune responses, cognitive deficits [13,16,17], emotional disorders [13,15] and elevated expression of amyloid precursor protein and β-amyloid in the brain [18]. Evidence indicates that cognitive deficits in SAMP8 can be observed as early as four months after birth, which is earlier than those in SAMR1 and that the deficits appear to be due to dysfunction of the neurobiological signaling mediated by some key proteins such as Ca^2+^/calmodulin-dependent kinase II (CaMKII), cyclic AMP responsive element binding protein (CREB) and N-methyl-D-aspartate receptor (NMDAR), which are important for synaptic plasticity [13,15,19,20]. Moreover, our previous study suggested that the VEGF/VEGFR2 signaling system in the brain is down-regulated in the SAMP8 animals and that the amelioration of cognitive deficits of SAMP8 implies the improvement of the system [13]. These features of SAMP8 provide a useful animal model for the investigation of the neurological and molecular biological basis for cognitive dysfunction caused by aging in humans.

This study investigates the effect of CTS on cognitive deficits in an animal aging model, namely SAMP8, with and without ischemic insult, to evaluate whether CTS can be used as an anti-dementia drug to treat aging-related cognitive deficits.

## Methods

### Animals

**Figure 1:**
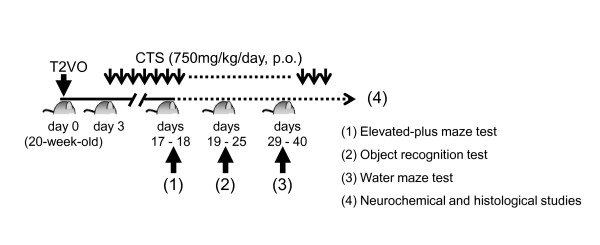
**Schematic drawing of the experimental schedule in this study**. Transient ischemic operation was conducted at day 0. From day 3, administration of CTS to the SAMP8 group was started.

Male SAMP8 and SAMR1 aged six weeks were obtained from SLC Inc. (Japan). Mice were housed in a laboratory animal room maintained at 25 ± 1°C with 65 ± 5% humidity on a 12-hour light/dark cycle (07:30 to 19:30). Animals were given food and water *ad libitum*. The present study was conducted in accordance with the Guiding Principles for the Care and Use of Animals (NIH publication #85-23, revised in 1985) and complied with the Helsinki Declaration [21]. The present study was also approved by the Institutional Animal Use and Care Committee of the University of Toyama. A detailed experimental schedule is described in Figure 1.

### Preparation and chemical profiling of CTS

CTS extract used in this study was purchased from Tsumura Co. (Japan) in the form of a spray-dried powder extract prepared according to the standardized extraction method of medicinal plants registered in the *Japanese Pharmacopoeia XV*. The CTS extract was from the same lot (Lot #202004-7010) used in a previous study [2]. This extract was prepared from a mixture of 3.0 parts *Uncariae Uncis cum Ramulus *(hooks and branch of *Uncaria rhynchophylla *MIQUEL), 3.0 parts *Aurantii Nobilis pericarpium *(peel of *Citrus unshiu *MARKOVICH), 3.0 parts *Pinelliae tuber *(tuber of *Pinellia ternate *BREITENBACH), 3.0 parts *Ophiopogonis tuber *(root of *Ophiopogon japonicus *KER-GAWLER), 3.0 parts Hoelen (sclerotium of *Poria cocos *WOLF), 2.0 parts *Ginseng radix *(root of *Panax ginseng *C.A. MEYER), 2.0 parts *Saphoshnikoviae radix et rhizoma *(root and rhizome of *Saposhnikovia divaricata *SCHISCHKIN), 2.0 parts *Chrysanthemi flos *(flower of *Chrysanthemum morifolium *RAMATULLE), 1.0 part *Glycyrrhizae radix *(root of *Glycyrrhiza uralensis *FISHER), 1.0 part *Zingiberis rhizoma *(rhizome of *Zingiber officinale *ROSCOE) and 5.0 parts *Gypsum fibrosum *(CaSO_4 _2H_2_O). The yield of the CTS extract was 16.1%.

To identify the chemical constituents of CTS, 3D-HPLC analysis was conducted as previously described [2,3]. Briefly, CTS (2.5 g, Tsumura, Japan) was filtered and then subjected to high-performance liquid chromatography (HPLC) analysis. HPLC equipment was controlled by an SLC-10A system controller (Shimadzu, Japan) with a TSKGELODS-80TS column (4.6×250 mm) (TOSOH, Japan), eluting with solvents (A) 0.05 M AcONH_4 _(pH3.6) and (B) CH_3_CN. A linear gradient (100% A and 0% B to 0% A and 100% B in 60 minutes) was used. The flow rate was controlled by an LC-10AD pump (Shimadzu, Japan) at 1.0 ml/min. The eluent from the column was monitored and processed with an SPD-M10A diode array detector (Shimadzu, Japan). The 3D-HPLC profiling data have been previously described [2,3]. For chemical profiling of CTS, liquid chromatography-mass spectrometry (LC-MS) analysis was performed with a Shimadzu LC-IT-TOF mass spectrometer (Japan) equipped with an ESI interface (Shimadzu, Japan). The ESI parameters were as follows: source voltage +4.5 kV, capillary temperature 200°C and nebulizer gas 1.5 l/min. The mass spectrometer was operated in positive ion mode scanning from *m/z *200 to 2000. A Waters Atlantis T_3 _column (2.1 mm i.d. × 150 mm, 3 m, USA) was used and the column temperature was maintained at 40°C. The mobile phase was a binary eluent of (A) 5 mM ammonium acetate solution and (B) CH_3_CN under the following gradient conditions: 0-30 minutes linear gradient from 10% to 100% B, 30-40 min isocratic at 100% B. The flow rate was 0.15 ml/min. Mass spectrometry data obtained from the extract were deposited in MassBank database [22] and stored with the pharmacological information on the extract in the Wakan-Yaku Database system [23], Institute of Natural Medicine, University of Toyama. The CTS extract used in this study was deposited at our institute (voucher specimen no. 20000005).

### Surgical operation for transient cerebral ischemia

Surgical operation to induce transient cerebral ischemia (T2VO) was conducted as previously described [3]. Briefly, at the age of 20 weeks, SAMP8 received transient occlusion of bilateral common carotid arteries for 15 minutes under pentobarbital-Na (50 mg/kg, i.p.) anesthesia. The animals that received the same operation without occlusion of carotid arteries served as sham-operated controls. From three days after the operation, the animals received daily administration of CTS (750 mg/kg, p.o.). The dose of CTS was selected on the basis of our previous studies using an animal model of cerebrovascular dementia [2,3].

### Behavioral assessment

#### Elevated plus maze test

The elevated plus maze is comprised of two open arms (22 × 8 cm) and two arms enclosed by high walls (22 × 8 × 17 cm), with an open roof, the two arms of each type being positioned opposite to each other as previously described [13]. The maze was set 60 cm above the floor. Each mouse was individually placed at the center of the maze facing one of the enclosed arms and allowed to explore the maze freely during a 5-minute observation period. Maze performance was video-recorded for later analysis. Time spent in open arms and the numbers of arm entries were analyzed as indices of emotional behavior using SMART^® ^ver. 2.5 (PanLab, SLU, Spain).

#### Learning and memory test

##### Nobel object recognition test (ORT)

ORT was conducted as previously described [2,13] with minor modifications. The apparatus consisted of a square arena (50 × 50 × 40 cm) made of polyvinyl chloride with gray walls and a black floor. The objects for recognition had visual patterns or visually different shapes to be discriminated. The ORT consisted of a sample phase trial and a test phase trial. In the sample phase trial, each mouse was first placed in the observation box where two identical objects, namely O1 and O2 (each of which was a 7.5 × 5.5 cm white cup), were placed separately and allowed to freely explore the arena for five minutes. The total time that the mouse spent exploring each of the two objects was measured and then the mouse was returned to the home cage. In the test phase trials performed ten minutes after the sample phase trials, one of the two objects was replaced by an identical copy (object F) and the other by a novel object (object N). Performance of the animals in this test was video-recorded for later analysis. In these trials, the exploration of an object was defined as directing the nose to the object at a distance of less than 2 cm according to previous reports [2,13] and the time spent exploring each of the two objects was analyzed with SMART^® ^ver. 2.5 (PanLab, SLU, Spain) with a tri-wise module to detect the head, center mass and base-tail. A discrimination index (DI) was calculated according to the following equation [2,13]:

$$\text{DI}=\left({\text{T}}_{\text{n}}-{\text{T}}_{\text{f}}\right)∕\left({\text{T}}_{\text{n}}+{\text{T}}_{\text{f}}\right)$$

where T_n _and T_f _represent the time spent exploring new and familiar objects respectively. The box arena and objects were cleaned with 75% ethanol between trials to prevent a build-up of olfactory cues.

##### Nobel object location test (OLT)

The OLT, which is a two-trial task with a sample phase trial and a test phase trial separated by an inter-trial interval, was conducted as previously reported [2,13]. The objects used in the sample phase trial were two black cones A1 and A2 (5 × 10 cm). Ten minutes after the sample phase trial, the test phase trial was conducted. In this trial, the objects were replaced by their identical copies, one of which was placed in the same position, whereas the other was moved to the adjacent corner, so that the two objects were in diagonally opposite corners. In the test phase trials, both objects were equally familiar to the animals, but one had changed location. The mice were exposed to the objects for five minutes. Performance of the animals was video-recorded and the total time spent exploring each of the two objects was analyzed as previously described.

##### Morris water maze test

The Morris water maze test was conducted with a circular pool (110 cm in diameter), a transparent platform (7 cm in diameter) and various extra maze cues surrounding the pool as previously described [2,3]. Twenty-nine days after surgery, the animals were subjected to a visible trial test (Visible 1) where the platform was made visible 1 cm above the water surface. One day after the visible trial, acquisition trials were performed daily for five days. The animals underwent four trials daily. In each training trial, the mouse was placed in the pool from one of the four start positions at 90° apart around the edge of the pool and then allowed to swim to the hidden transparent platform (7 cm in diameter). If the mouse had not found the platform during a 60 s period, it was placed onto the platform by the experimenter. The mouse was allowed to remain on the platform for 10 s before being placed in an opaque high-sided plastic chamber for 60 s. The next trial was then performed. Water maze behavior of each mouse was video-recorded for later analysis. In each trial, the latency to reach the platform (escape latency), distance covered and average swim speed were analyzed *via *a video capture and image analysis system (SMART^® ^system, PanLab, SLU, Spain). The daily trial data of each animal were averaged and expressed as a block of four trials before statistical analysis. One day after the last acquisition trials, a single 60 s probe trial was run in which the platform was removed from the pool. The time spent in each of the four imaginary quadrants of the pool was recorded and analyzed with the SMART^® ^system.

### Quantitative real-time PCR

Quantitative real-time PCR was conducted as previously described [13]. Briefly, the animals were decapitated after completing the behavioral experiments. The cerebral cortices were dissected out and kept at -80°C until use. Total RNA was extracted from the cortex with Sepazol^® ^(Nacalai Tesque, Japan) according to the manufacturer's instructions. First-strand cDNA was synthesized with oligo (dT) primers and M-MLV reverse transcriptase^® ^(Invitrogen, USA) and was used as a template for real-time PCR. Quantitative real-time PCR was carried out with Fast SYBR Green Master Mix and the StepOne Real-time PCR System^® ^(Applied BioSystem, USA). The following primer sets of BDNF and β-actin were designed by Perfect Real Time support system (Takara Bio Inc., Japan): BDNF (NM_007540): 5'-AGCTGAGCTGTGTGACAGT-3' (forward) and 5'-TCCATAGTAAGGGCCCGAAC-3' (reverse); β-actin (NM_007393): 5'-CATCCGTAAAGACCTCTATGCCAAC-3' (forward) and 5'-ATGGAGCCACCGATCCACA-3' (reverse). Melting curve analysis of each gene was performed every time after amplification. In all reactions, β-actin mRNA was used as a control to which the results were normalized. Standard curves of the log concentration of each gene *vs*. cycle threshold were plotted to prove inverse linear correlations. The correlation coefficients for standard curves of target genes were 0.9965 to 0.998.

### Western blotting analysis

Western blotting was performed as previously described with minor modifications [13,24]. Briefly, tissue samples were taken from the cortices and homogenized in lysis buffer TissueLyser^® ^(Qiagen, Japan) consisting of 50 mM Tris HCl buffer (pH7.4), 150 mM NaCl, 0.5% sodium deoxycholate, 1% (v/v) NP-40, 0.1% (v/v) sodium dodecyl sulfate (SDS), 150 mM NaF, 8.12 μg/ml aprotinin, 2 mM sodium orthovanadate, 10 μg/ml leupeptin, and 2 mM phenylmethylsulfonyl fluoride. The lysate samples were then centrifuged at 10,000 rpm (9200 *g*, Kubota 3740, Kubota Co., Japan) at 4°C for five minutes. The protein concentration of the supernatant was determined with a BCA™ protein assay kit (Thermo Scientific, USA). Each protein sample was mixed with Laemmli sample buffer and denatured at 95°C for three minutes. The proteins (20 μg) from each sample were electrophoresed on 5-12% sodium dodecyl sulfate polyacrylamide gel (SDS-PAGE) and then electro-blotted onto a polyvinylidene difluoride membrane (Bio-rad Laboratory, USA). The membranes were incubated in a 5% non-fat milk-containing wash buffer (Nacarai Tesque, Japan) (50 mM Tris HCl pH7.5, 150 mM NaCl and 0.1% Tween 20) for one hour at room temperature. They were then probed with anti-NMDAR1 rabbit polyclonal antibody (1:1000 dilution) and anti-phospho-NMDAR1 (p-NMDAR1) (Ser896) rabbit polyclonal antibody (1:1000 dilution), anti-CaMKIIα (A-1: sc-13141) mouse monoclonal antibody (1:1000 dilution), anti-phospho-CaMKII (p-CaMKII) (Thr286) rabbit polyclonal antibody (1:1000 dilution) (Cell Signaling Technology, USA), anti-CREB (48H2) rabbit monoclonal antibody (1:1000 dilution), anti-phospho-CREB (p-CREB) (Ser133) rabbit monoclonal antibody (1:1000 dilution), anti-BDNF (Tyr951) rabbit polyclonal antibody (1:500 dilution), anti-VEGF (A-20: sc-152) rabbit polyclonal antibody (1:1000 dilution) (Santa Cruz Biotechnology, USA), and anti-VEGFR2 (Ab-951) rabbit polyclonal antibody (1:1000 dilution) (Signalway Antibody, USA) and anti-glyceraldehyde-3-phosphate dehydrogenase (GAPDH) mouse monoclonal antibody (1:2000 dilution) (Chemicon, USA) at 4°C for 24 hours. After the membranes were rinsed in wash buffer without non-fat milk, the blots were incubated with anti-mouse or anti-rabbit secondary antibodies linked with horseradish peroxidase (Dako Cytomation EnVision + System-HRP-labeled Polymer) (Dako Cytomation Inc., USA) according to the manufacturer's instructions. The quantity of immunoreactive bands was detected by an enhanced chemiluminescence method (ImmobilonTM Western Chemiluminescent HRP Substrate) (Millipore, USA) and imaged with Lumino Image Analyzer LAS-4000 (Fuji Film, Japan). The signal intensity was normalized by comparing with their expression levels in treatment-naïve control mice. Each membrane was re-probed with Blot Restore Membrane Rejuvenation Kit (Chemicon, USA). The band images were analyzed with VH-H1A5 software (Keyence, Japan).

### Immunohistochemistry

CTS administration-induced changes in expression levels of VEGF and PDGF-A in the cerebral cortex of sham-SAMP8 and T2VO-SAMP8 were also examined with immunohistochemical analysis. Briefly, the animals were fixed by intracardiac perfusion of 4% paraformaldehyde in phosphate buffered saline (PBS) under pentobarbital anesthesia. Brains were post-fixed with 4% paraformaldehyde overnight at 4°C. A series of 5 μm coronal sections from different brain regions including cerebral cortex and hippocampus were obtained. The paraffin-embedded specimens were deparaffinized in xylene and dehydrated with ethanol. Endogenous peroxidase was blocked with 0.1% hydrogen peroxide-methanol for 30 minutes at room temperature. Washed with Tris-buffered saline (TBS), the specimens were incubated in a microwave oven (95°C, 750 W; MF-2; Nissin, Japan) in target retrieval solution (Dako, Denmark) for 15 minutes and then washed with distilled water and TBS. Nonspecific binding was blocked by treatment with a special blocking reagent (Dako, Denmark) for 15 minutes. The specimens were challenged with 1:200 dilution of anti-VEGF or anti-PDGF-A antibody and then incubated in a moist box at 4°C overnight. Washed with TBS, the specimens were incubated with a peroxidase-conjugated anti-rabbit IgG polymer (Envision-PO for Rabbit; Dako, Denmark). After three washes in TBS, a reaction product was detected with 3,3'-diaminobenzidine tetrahydrochloride (0.25 mg/ml) and hydrogen peroxide solution (0.01%). Counter-stained with hematoxylin, the sections were rinsed, dehydrated, and covered. Also included in each staining run were negative controls in which the primary antibody was omitted. The images were captured with a microscope (AX-80, Olympus, Japan).

### Statistical analysis

Statistical analysis of the data was conducted according to Curran-Everett and Benos [25]. All data are expressed as mean ± standard deviation (SD). Statistical analyses of the behavioral data comprised paired and unpaired Student's t-tests, a two-way analysis of variance (ANOVA), or two-way repeated measures ANOVA followed by the Student-Newman-Keuls test, as appropriate. The mRNA and protein expression levels were evaluated with Student's t-test or a two-way analysis of variance (ANOVA) followed by the Student-Newman-Keuls test. The analysis was conducted using SigmaStat^® ^ver 3.5 (SYSTAT Software Inc., USA). Differences of *P *< 0.05 were considered significant.

## Results

### Behavioral studies

#### Effect of CTS on emotional disorder of sham- and T2VO-SAMP8 in the elevated plus maze test

**Figure 2:**
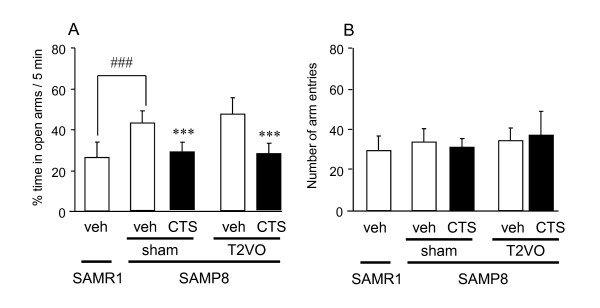
**Effects of CTS administration on elevated plus maze performance of SAMP8 with and without ischemic insult**. The 20-week-old SAMP8 received sham operation or transient occlusion of common carotid arteries (T2VO) for 15 minutes on day 0 and then received oral administration of water (vehicle) or 750 mg/kg CTS once daily during an experimental period. The elevated plus maze test was conducted on days 17 and 18 after ischemic operation. Each datum represents the mean ± SD (9-10 mice per group). The proportion of time spent in open arms (A) and the number of total arm entries (B) were calculated. The data are expressed as the mean ± SD ^###^*P *< 0.001 vs. vehicle-treated SAMR1 group (t-test). ***P < 0.001 vs. vehicle-treated sham- or T2VO-SAMP8 groups (two-way ANOVA).

The elevated plus maze test was conducted to elucidate the effect of CTS on emotional deficits of SAMP8 that had received sham or T2VO operation. The sham- and T2VO-SAMP8 treated with vehicle spent a significantly longer time exploring the open arms than the SAMR1 controls (t = -5.468, df = 17, *P *< 0.001, t-test). The administration of CTS (750 mg/kg per day, p.o.) to sham- and T2VO-SAMP8 reduced the proportion of time spent in open arms by these animal groups [F_drug treatment _(1,34) = 76.639, *P <*0.001, two-way ANOVA]. No significant difference in the effect of CTS and T2VO operation on total arm entries was observed between sham- and T2VO-SAMP8 [F_drug treatment _(1,34) = 0.00021, *P *= 0.989 and F_operation _(1,34) = 1.851, *P *= 0.183, two-way ANOVA] (Figure 2).

#### CTS amelioration of non-spatial cognitive deficits of sham- and T2VO-SAMP8 in ORT

**Figure 3:**
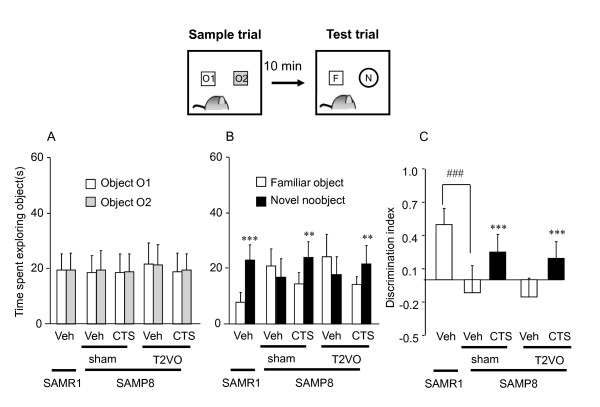
**Effects of CTS on object discrimination performance of SAMP8 with and without ischemic insult in the object recognition test (ORT)**. The object recognition test was conducted on days 19-21 after T2VO operation. Each datum represents the mean ± SD (ten mice per group). (A) The data from the sample trials of the ORT. The animal was placed into the arena where two identical sample objects made of glass (objects O1 and O2) were placed in two adjacent corners of the arena and was allowed to explore for five minutes. There was no significant difference in performance in the sample phase trial among the groups. (B) The data from the test phase trials conducted ten minutes after the sample phase trials. In the test phase trials, the time animals spent exploring a familiar object or a new object was measured during a 5-minute observation period. ***P < 0.001 and **P < 0.01 vs. the time spent exploring a familiar object (paired t-test). (C) Discrimination index (DI) in the ORT. DI was calculated as described in the text. ^###^*P *< 0.001 vs. vehicle-treated SAMR1 group (t-test). ***P < 0.001 vs. vehicle-treated SAMP8 group (two-way ANOVA).

The non-spatial cognitive performance of sham- and T2VO-SAMP8 was elucidated by the ORT. The sample phase trials of the ORT revealed no differences in total time spent exploring two identical objects between SAMR1 and sham-SAMP8 [t = 0.206, df = 18, *P *= 0.839]. Moreover, there was no significant interaction between T2VO operation and CTS administration in terms of performance of SAMP8 groups in the sample phase trials [F_operation × CTS treatment _(1,36) = 0.285, *P *= 0.597, two-way ANOVA] (Figure 3A). However, in the test phase trials, SAMR1 spent a significantly longer time exploring a novel object than exploring a familiar object [t = 9.05, df = 9, *P *< 0.001; paired t-test], indicating preference for the novelty. By contrast, sham- and T2VO-SAMP8 showed no preference for the novel object [sham-SAMP8: t = -1.263, df = 9, *P *= 0.238] or still spent a longer time exploring the familiar object than the novel object [T2VO-SAMP8: t = -3.413, df = 9, *P *= 0.008, paired t-test]. Treatment of sham- and T2VO-SAMP8 with CTS (750 mg/kg/day, p.o.) normalized novel object recognition behavior of these animal groups which spent a significantly longer time on the novel object than on the familiar object [CTS-treated sham-SAMP8: t = 4.094, df = 9, *P *= 0.003; CTS-treated T2VO-SAMP8: t = 4.136, df = 9, *P *= 0.003, paired t-test] (Figure 3B). Analysis of the DI values also revealed that the CTS administration improved the object recognition deficit of the SAMP8 (sham and T2VO) group [F_CTS treatment _(1,36) = 37.061, *P *< 0.001, two-way ANOVA] and that no significant interaction was observed between T2VO operation and CTS treatment in the performance of SAMP8 groups [F_operation × CTS treatment_(1,36) = 0.0307, *P *= 0.862]. Moreover, compared with SAMR1, the DI value of the vehicle-treated SAMP8 was significantly decreased (t = 6.845, df = 18, *P *< 0.001, t-test) (Figure 3C).

#### Effect of CTS on special cognitive performance in OLT and water maze test

##### Object location test

**Figure 4:**
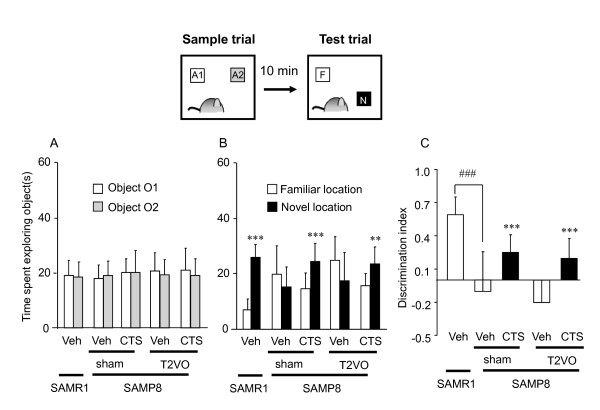
**Effects of CTS on object discrimination performance of SAMP8 with and without ischemic insult in the object location test (OLT)**. The OLT was conducted on days 23-25 after T2VO operation. Each datum represents the mean ± SD (ten mice per group). (A) The data from the sample trials of the OLT. The animal was placed into the arena where two identical sample objects made of glass (objects A1 and A2) were placed in two adjacent corners of the arena and was allowed to explore for five minutes. There was no significant difference in performance in the sample phase trial among the groups. (B) The data from the test phase trials conducted ten minutes after the sample phase trials. In the test phase trials, the time animals spent exploring an object placed in the familiar and a new location was measured during a 5-minute observation period. ***P < 0.001 and **P < 0.01 vs. the time spent exploring the familiar location (paired t-test). (C) Discrimination index (DI) in the OLT. DI was calculated as described in the text. ^###^*P *< 0.001 vs. vehicle-treated SAMR1 group (t-test). ***P < 0.001 vs. vehicle-treated sham- or T2VO-SAMP8 groups (two-way ANOVA).

In the OLT, analysis of the sample phase trials revealed no significant differences in the total exploration time spent on identical objects between SAMR1 and sham-SAMP8 [t = 0.192, df = 18, *P *= 0.85, t-test ] or among SAMP8 groups [F_operation × CTS treatment_(1,36) = 0.210, *P *= 0.650] (Figure 4A). In the test phase trials, the SAMR1 and CTS-treated SAMP8 groups clearly showed a preference for an object placed in a novel location compared with an object placed in a familiar location [SAMR1: t = -10.803, df = 9, *P <*0.001; CTS-treated sham-SAMP8: t = -5.806, df = 9, *P *< 0.001; CTS-treated T2VO-SAMP8: t = -3.359, df = 9, *P *= 0.008, paired t-test]. By contrast, the sham-SAMP8 and T2VO-SAMP8 groups treated with water vehicle were unable to discriminate a novel location from a familiar location or spent more time exploring the object placed in a familiar location [sham-SAMP8: t = 0.985, df = 9, *P *= 0.350; and T2VO-SAMP8: t = 3.109, df = 9, *P *= 0.013, paired t-test] (Figure 4B). Two-way ANOVA of the DI among the SAMP8 groups revealed a significant effect of CTS treatment [F_CTS treatment_(1,36) = 24.961, *P *< 0.001]. CTS administration significantly reversed DI values to the levels of the SAMP8 control group. Moreover, compared with SAMR1, the DI of the vehicle-treated SAMP8 was significantly decreased (t = 5.635, df = 18, *P *< 0.001, t-test) (Figure 4C).

##### Morris water maze test

**Figure 5:**
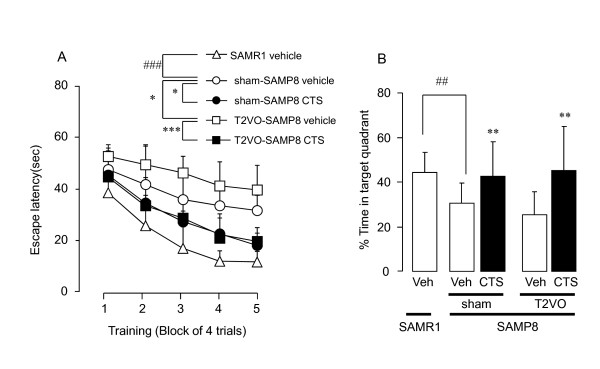
**CTS administration-induced amelioration of impaired water maze performance of SAMP8 mice with and without ischemic insult**. The water maze test was conducted on days 29-40 after T2VO operation. (A) Learning performance of the animals elucidated in the training test. Each data point indicates the mean escape latency ± SD for 6-10 animals in each group. ^###^*P *< 0.001, *P < 0.05, and ***P < 0.001 (two-way ANOVA for repeated measurement). (B) Memory retrieval performance elucidated in the probe test. The test was conducted 24 hours after the last training trials. Each datum represents the mean of time spent in the target quadrant ± SD ^##^*P *< 0.01 compared with vehicle-treated SAMR1 group. **P < 0.01 compared with respective vehicle-treated sham- or T2VO-SAMP8 group (two-way ANOVA).

In order to test whether T2VO exacerbates spatial memory deficits of SAMP8, we used the water maze test based on a hippocampus-dependent learning paradigm (Figure 5). Each animal group could learn the location of the submerged platform following repeated daily training [F_training_(4,56) = 28.377, *P *< 0.001, two-way repeated measures ANOVA] but the escape latency of the sham-SAMP8 vehicle control group was significantly longer than that of SAMR1 control [F_animal × training_(4,56) = 2.921, *P *= 0.029, two-way repeated measures ANOVA]. Moreover, the T2VO-SAMP8 mice displayed significantly longer latencies than the sham-SAMP8 group to find a platform [F_operation_(1,13) = 5.241, *P *= 0.039, two-way repeated measures ANOVA] in the training trials. We also examined the effect of CTS on spatial cognitive performance of the sham- and T2VO-SAMP8 in the water maze test. Daily treatment of sham-SAMP8 and T2VO-SAMP8 mice with 750 mg/kg CTS resulted in a significant decrease in escape latencies of these animal groups [sham-SAMP8: F_CTS treatment_(1,11) = 7.076, *P *= 0.022; T2VO-SAMP8: F_CTS treatment_(1,17) = 59.484, *P *< 0.001, two-way repeated measures ANOVA] (Figure 5A).

In the probe test conducted one day after a 5-day training period, swimming time of the sham-SAMP8 control in the target quadrant where the platform was placed during training was significantly shorter than that of the SAMR1 [t = 3.009, df = 14, *P *= 0.009, t-test]. The sham- and T2VO-SAMP8 groups treated with daily administration of CTS (750 mg/kg) spent a longer time swimming in the target quadrant than those of the vehicle-treated sham- and T2VO-SAMP8 groups [F_CTS treatment_(1,28) = 8.599, *P *= 0.007, two-way ANOVA] but no significant interaction was detected between the T2VO operation and CTS treatment in the SAMP8 groups [F_operation × CTS treatment_(1,28) = 0.502, *P *= 0.484, two-way ANOVA] (Figure 5B).

### Neurochemical studies

#### CTS reverses synaptic plasticity-related signaling down-regulated in the cerebral cortex of sham- and T2VO-SAMP8

**Figure 6:**
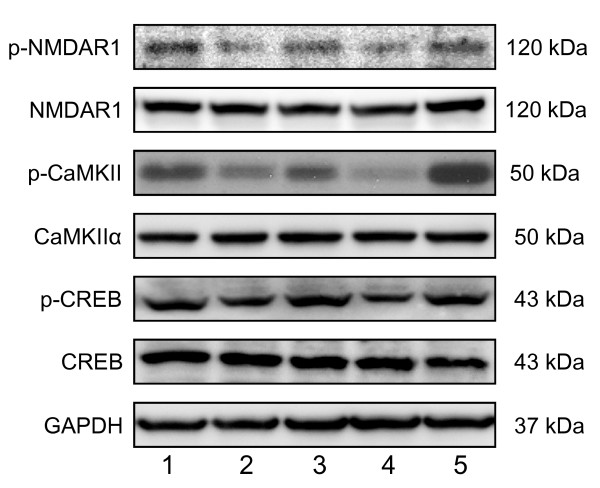
**Effects of CTS on expression levels of p-NMDAR1, NMDAR1, p-CaMKII, CaMKIIα, p-CREB, CREB, and GAPDH in the cerebral cortex of SAMP8 with and without ischemic insult**. Typical photos indicating the expression levels of each factor in the cerebral cortex of vehicle-treated SAMR1 control (lane 1), vehicle-treated sham-SAMP8 (lane 2), CTS (750 mg/kg/day)-treated sham-SAMP8 (lane 3), vehicle-treated T2VO-SAMP8 (lane 4), and CTS-treated T2VO-SAMP8 group (lane 5). After completing the behavioral studies, the animals were decapitated and proteins were extracted from the cerebral cortices in each animal group.

In order to understand the molecular mechanism(s) underlying CTS-induced improvement of cognitive deficits in the sham- and T2VO-SAMP8, we examined the effects of CTS on synaptic plasticity-related signaling by measuring phosphorylation activities of NMDAR1, CaMKII and CREB phosphorylation in the cortex areas (Figure 6). Compared with SAMR1, the sham-SAMP8 groups had significantly reduced levels of p-NMDAR1 [t = 2.643, df = 8, *P *= 0.030, t-test], p-CaMKII [t = 2.746, df = 8, *P *= 0.025, t-test] and p-CREB (t = 4.677, df = 8, *P *= 0.002, t-test). CTS administration to the sham-SAMP8 and -T2VO groups significantly reversed the decreased levels of p-NMDA [F_CTS treatment _(1,16) = 14.326, *P *= 0.002, two-way ANOVA], p-CaMKII [F_CTS treatment_(1,16) = 15.952, *P *= 0.001, two-way ANOVA] and p-CREB [F_CTS treatment_(1,16) = 11.262, *P *= 0.004, two-way ANOVA] in these animal groups. However, no significant difference in the expression levels of NMDAR1, CaMKIIα and CREB was observed between the SAMR1 and sham-SAMP8 or among the SAMP8 groups.

**Figure 7:**
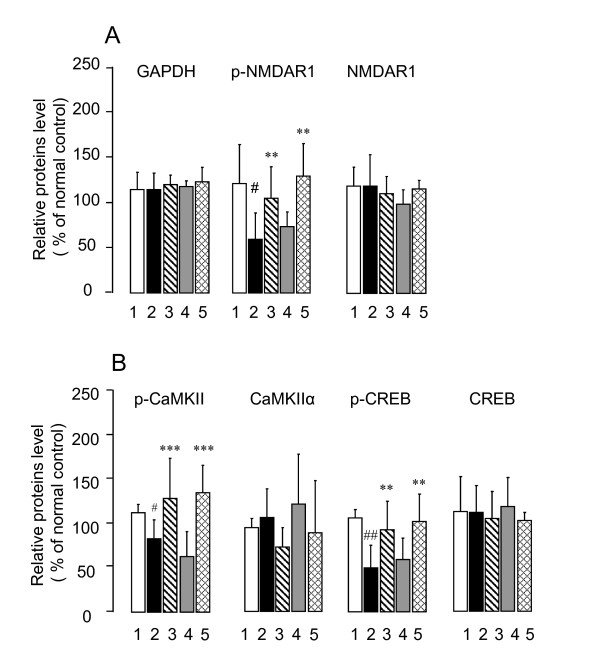
**Quantitative comparisons of CTS-induced changes in expression levels of p-NMDAR1, NMDAR1, p-CaMKII, CaMKIIα, p-CREB, CREB, and GAPDH in the cerebral cortex of SAMP8 with and without ischemic insult**. The data are expressed as the percentage of the value obtained from naïve control SAMR1 mice. Each data column represents the mean ± SD obtained from 4-5 brain samples. ^#^*P *< 0.05 or ^##^*P *< 0.01 vs. compared with vehicle-treated SAMR1 group (t-test). ***P < 0.001, **P < 0.01 vs. respective vehicle-treated sham- or T2VO-SAMP8 group (two-way ANOVA).

**Figure 8:**
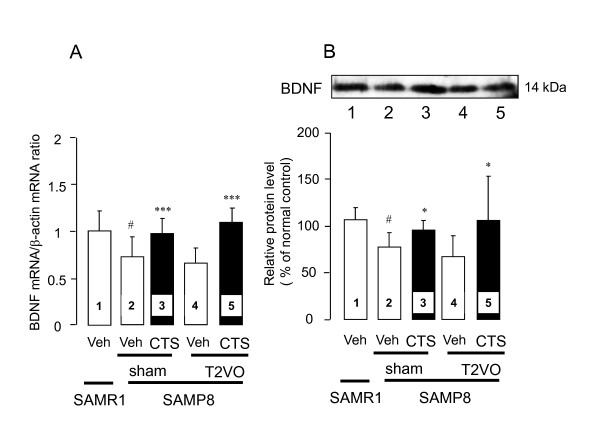
**Effects of CTS on BDNF mRNA and protein expression in the cerebral cortex**. After completing the behavioral studies, we decapitated the animals and prepared the protein and total RNA from the cerebral cortices. Real-time PCR analysis of BDNF mRNA (A) and BDNF protein (B) expression in vehicle-treated SAMR1 (lane 1) and vehicle-treated sham-SAMP8 (lane 2), CTS (750 mg/kg/day)-treated sham-SAMP8 (lane 3), vehicle-treated T2VO-SAMP8 (lane 4), and CTS-treated T2VO-SAMP8 (lane 5). Each data column represents the mean ± SD obtained from 5 brain samples. ^#^*P *< 0.05 vs. vehicle-treated SAMR1 group (t-test). *P < 0.05 and ***P < 0.001 vs. respective vehicle sham- or T2VO-SAMP8group (two-way ANOVA).

We also measured the expression levels of BDNF gene transcript and BDNF protein which is a functional molecule downstream of the transcriptional activity of CREB, *via *CREB phosphorylation, in the brain (Figure 7). In contrast to the SAMR1, the SAMP8 had significantly reduced levels of BDNF mRNA (t = 3.238, df = 8, *P *= 0.012, t-test) and its protein (t = 3.964, df = 8, *P *= 0.011, t-test) in the cerebral cortex. However, daily administration of CTS to sham- and T2VO-SAMP8 significantly reversed the decreases in the expression levels of BDNF mRNA [F_CTS treatment_(1,16) = 19.746, *P *< 0.001, two-way ANOVA] and BDNF protein [F_CTS treatment_(1,16) = 5.135, *P *= 0.038, two-way ANOVA] in these animal groups (Figure 8). The extent to which CTS reversed the expression level of the BDNF mRNA was not significantly different between the sham- and T2VO-SAMP8 groups. Western blotting analysis also confirmed that the amelioration of the transcription process of BDNF mRNA in sham- and T2VO-SAMP8 animals occurred after the daily CTS administration.

#### Effects of CTS on the expression levels of VEGF and PDGF, angiogenic and neurotrophic factors, in the cerebral cortex of sham- and T2VO-SAMP

**Figure 9:**
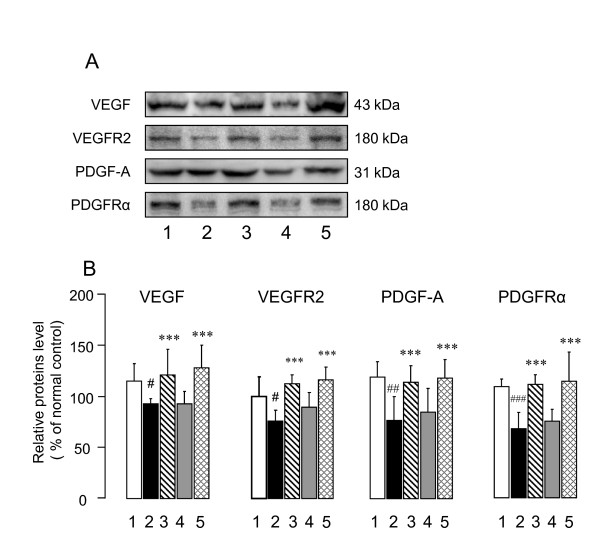
**Effects of CTS on VEGF/VEGFR2 and PDGF-A/PDGFRα expression in the cerebral cortex of SAMP8 with and without ischemic insult**. After completing the behavioral studies, we decapitated the animals and extracted the total protein from the cerebral cortices as described in the text. (A) Typical photos indicating the expression levels of VEGF, VEGFR2, PDGF, and PDGFRα in the cerebral cortex of vehicle-treated SAMR1 control (lane 1), vehicle-treated sham-SAMP8 (lane 2), CTS (750 mg/kg/day)-treated sham-SAMP8 (lane 3), vehicle-treated T2VO-SAMP8 (lane 4), and CTS-treated T2VO-SAMP8 group (lane 5). (B) Quantitative comparisons of each factor among different animal groups were conducted as described in the text. The data are expressed as the percentage of the value obtained from naïve control SAMR1 mice. Each data column represents the mean ± SD obtained from five brain samples. ^#^*P *< 0.05, ^##^*P *< 0.01 and ^###^*P *< 0.001 vs. vehicle-treated SAMR1 group (t-test). ***P < 0.001 vs. respective vehicle-treated sham- or T2VO-SAMP8group (two-way ANOVA).

**Figure 10:**
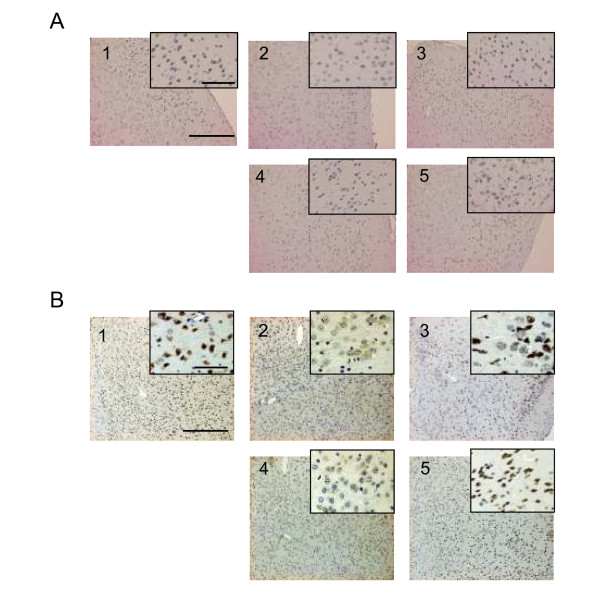
**Immunohistochemical evaluation of the effect of CTS on aging-induced decrease in VEGF (A) and PDGF-A (B) in the prefrontal cortex region**. (1) vehicle-treated SAMR1, (2) vehicle-treated sham-SAMP8, (3) CTS (750 mg/kg/day)-treated sham-SAMP8, (4) vehicle-treated T2VO-SAMP8 and (5) CTS-treated T2VO-SAMP8 group. Scale bars in each photo and inset represent 100 μm and 50 μm respectively.

Since the VEGF/PDGF family has angiogenic and neurotrophic roles in the central nervous system and its level declines with aging [26-28], we evaluated the effects of the CTS treatment on the VEGF/VEGFR2 and PDGF-A/PDGFRα systems in the cerebral cortex. Western blotting analysis (Figure 9) revealed that, compared with SAMR1, the sham-SAMP8 groups showed reduced levels of VEGF (t = 2.829, df = 8, *P *= 0.022, t-test), VEGFR2 (t = 2.328, df = 8, *P *= 0.048, t-test), PDGF-A (t = 3.41, df = 8, *P *= 0.009, t-test) and PDGFR-α (t = 5.419, df = 8, *P *< 0.001, t-test). However, the CTS administration significantly up-regulated the expression levels of VEGF [F_drug _(1,16) = 16.008, *P *= 0.001, two-way ANOVA], VEGFR2 [F_CTS treatment_(1,16) = 35.591, *P *< 0.001, two-way ANOVA], PDGF-A [F_drug _(1,16) = 15.118, *P *= 0.001, two-way ANOVA] and PDGFRα [F_CTS treatment_(1,16) = 26.571, *P *< 0.001, two-way ANOVA] in the sham- and T2VO-SAMP8 groups. No significant interaction between the ischemic operation and CTS administration was observed [VEGF: F_operation×CTS treatment_(1,16) = 0.244, *P *= 0.628; VEGFR2: F _operation×CTS treatment_(1,16) = 0.885, *P *= 0.361; PDGF-A: F _operation×CTS treatment_(1,16) = 0.0713, *P *= 0.793; PDGFRα: F _operation×CTS treatment_(1,16) = 0.0576, *P *= 0.813]. Immunohistochemical experiments conducted in this study (Figure 10) also revealed that the cortical expression levels of VEGF and PDGF-A in the vehicle-treated sham- and T2VO-SAMP8 groups were clearly lower than those in the vehicle-treated SAMR1 and that the CTS-treated SAMP8 groups had expression levels of these factors comparable to those in the vehicle-treated SAMR1 group.

## Discussion

This study aimed to clarify whether CTS has the therapeutic potential for aging-related cognitive deficits. To this end, we investigated the effects of CTS on emotional and cognitive deficits in an animal model of aging, namely SAMP8, with and without ischemic insult. The results have demonstrated that daily administration of CTS ameliorates both emotional and cognitive deficits of SAMP8 with and without ischemic insult and suggested that the effect on the deficits is attributable to the recovery of neuroplasticity-related neuronal signaling and the VEGF/PDGF signaling systems deteriorated by aging.

### CTS-induced improvement of emotional deficits of SAMP8

The elevated plus maze test conducted in this study revealed that sham- and T2VO-SAMP8 groups exhibited significantly reduced anxiety-like behavior compared with the control animal group, namely SAMR1, and that this abnormal emotional behavior was attenuated by CTS. The reduced anxiety level observed in SAMP8 animals matches the previous findings reported by Miyamoto *et al*. [29] and our group [13]. This characteristic behavioral symptom observed in SAMP8 animals provides potential utility of this animal strain as a model to investigate aging-related symptoms of patients with dementia [29]. Indeed, it has been reported that the emotional deficit of SAMP8 occurs in an aging-related manner and is, to some extent, relevant to dysfunction of central noradrenergic function in this animal strain and that long-term and continuous infusion of thyrotropin-releasing hormone (TRH) appears to be useful for treatment of the emotional disorders and memory disturbance caused in SAMP8 [29]. Therefore, it is of interest to note in this study that CTS administration during an experimental period could ameliorate emotional deficits of SAMP animals with and without ischemic insult. Taken together with aforementioned previous findings, the present results allow us to speculate that CTS administration improves emotional and cognitive deficits under a mechanism similar to that of TRH. The exact mechanism underlying the effect of CTS on emotional deficits of SAMP8 requires further investigation.

### Ameliorative effects of CTS on cognitive deficits caused by aging with ischemic factor

Cognitive deficit is one of the prominent symptoms caused by deterioration of brain function [13,19]. Moreover, aging and ischemic insults are risk factors implicated in the pathophysiology of cognitive deficits in patients with dementia. Therefore, in this study, we first evaluated the cognitive performance of SAMP8 mice with and without ischemic insult to create an animal model of dementia involving these risk factors. The results have demonstrated that SAMP8 animals with and without T2VO exhibit severely impaired learning and memory performance in the tests used to evaluate non-spatial (ORT) and spatial cognitive performance (OLT and water maze test). These findings agree with previous studies using the same strain of mice [13,30] and a mouse model of cerebrovascular dementia [2,3,31]. Moreover, the present findings have also revealed that, although T2VO has no effect on impaired short-term memory performance of SAMP8 in the ORT or OLT, it significantly exacerbates spatial reference memory performance. The reason for this different susceptibility of cognitive performances of SAMP8 to T2VO operation between the object recognition-type tests (ORT and OLT) and the water maze test is unclear. It may be due to a difference in difficulty to learn these tasks and/or to the extent to which an aging factor of SAMP8 affects cognitive ability used in these tests. Nevertheless, it is conceivable that SAMP8 with T2VO can also be used as an appropriate model for aging-related dementia.

It should be noted that CTS administration ameliorates not only emotional disorders but also learning and memory deficits observed in SAMP8 animals with and without ischemic insult. We have previously reported that CTS treatment or the cholinesterase inhibitor tacrine improves spatial and non-spatial cognitive deficits caused by chronic cerebral hypoperfusion in mice [2,31], indicating a therapeutic potential of CTS for cerebrovascular dementia. Taken together, the present results indicate that CTS administration exhibits a beneficial effect on cognitive deficits attributable not only to cerebrovascular impairments but also to an aging factor, providing further pharmacological evidence for the utility of CTS as a potential anti-dementia drug.

### Improvement of neuroplasticity-related signaling by CTS administration

To obtain evidence at the molecular level for CTS-induced improvement of cognitive deficits in SAMP8 with and without ischemic insult, we analyzed an important molecular biological feature of learning and memory in the brain, namely the expression of signaling proteins relevant to neuroplasticity. Lines of evidence have demonstrated that glutamatergic systems, such as NMDAR, are one of the molecular bases underlying learning and memory [32] and that phosphorylation of some key proteins such as NMDAR, CAMKII and CREB triggered by glutamate receptor stimulation is a molecular mechanism underlying neuroplasticity [19]. Indeed, activation of NMDA-type glutamate receptors increases the intracellular Ca^2+ ^level *via *the glutamate-gated Ca^2+^/Na^+ ^channels on neuronal membranes, thereby activating calmodulin and other Ca^2+^-dependent enzymes. This signaling elicits the phosphorylation of an NMDAR (GluR1) subunit protein at Ser896 in the hippocampal and cortical glutamatergic synapses *via *the activation of protein kinase C. Moreover, autophosphorylation of CaMKII triggered by intracellular Ca^2+^-dependent activation of calmodulin is reportedly implicated in the conversion of short-term memory to long-term memory. This process appears to potentiate cognitive function by affecting a BDNF system [33]. CREB, one of the nuclear transcription factors targeted by protein kinases A and/or C, also plays an important role in memory formation in a variety of cognitive tasks involving different brain structures [34]. Its phosphorylated form, p-CREB, is implicated in the transcription of late downstream genes encoding proteins essential for learning and memory such as structural proteins, signaling enzymes or neurotrophic/growth factors including BDNF [5]. In the present study, we analyzed these factors in the cortex since the cortex plays an important role in object recognition performance [35]. As shown in Figures 6 and 7, the vehicle-treated sham- and T2VO-SAMP8 groups had significantly reduced levels of p-NMDAR1, p-CaMKII, and p-CREB in the cortex compared with the age-matched SAMR1 control. In addition, expression levels of BDNF mRNA and its protein were decreased in these animal groups. These findings are consistent with our previous report [13]. Together, our findings indicate that dysfunction of signal transduction mechanisms related to memory formations is also caused in SAMP8, supporting the idea that the reduction of these phosphorylated proteins is linked to and/or represents the impaired performance of SAMP8 in the memory tasks.

Importantly, daily administration of CTS significantly reversed an aging-induced decrease in phosphorylation of NMDAR1, CaMKII, and CREB, as well as the expression of BDNF mRNA and its protein in the cerebral cortex. The detailed mechanism underlying these actions of CTS in SAMP8 groups is unclear. However, considering the close linkage of these factors to the BDNF protein and mRNA expressions in the brain, it is likely that the ameliorative effects of CTS on cognitive deficits caused in SAMP8 with and without ischemic insult are in part due to the improvement of neuronal signaling mediated by the glutamate receptor including an NMDAR subtype.

We previously reported on results of a study using an animal model of cerebrovascular dementia that the anti-dementia effect of CTS is in part mediated by the enhancement of central cholinergic function, particularly M_1 _muscarinic receptor stimulation [2,3,31]. Together, the present findings raise the possibility that CTS-induced reversal of impaired neurosignaling system in the brain of the SAMP8 groups is one of the outcomes resulting from enhancement of central cholinergic mechanisms by CTS. This idea is supported by lines of evidence. Endogenous acetylcholine reportedly exhibits a facilitatory role in the NMDA receptor function *via *M1 muscarinic receptors in the brain [36]. Moreover, evidence shows that the M_1 _receptor-mediated cognition behavior is mediated by neurosignaling pathways including the CREB phosphorylation and BDNF expression [37].

It has been demonstrated that aging process leads to an imbalance between oxidative damage and antioxidative defense system, and that most aging-induced physiological changes are due to molecular and cellular damage caused by free radicals [38]. In fact, reactive oxygen species (ROS) play a role in many neurodegenerative diseases including Alzheimer's disease [5,39,40] and ROS are accumulated in the blood and brain in the aging process, leading to the learning and memory deficits observed in SAMP8 [40]. Therefore, it is likely that, in this study, CTS administration attenuated the elevated level of oxidative stress in the brain, thereby suppressing cognitive impairment caused by oxidative brain damage in SAMP8. This possibility seems plausible because previous reports from our laboratory demonstrated that CTS effectively inhibited an oxidative stress-related process *via *enhancing antioxidant enzyme activity and scavenging ROS [41].

### A possible role of the VEGF and PDGF systems in the anti-dementia effects of CTS in SAMP8

One significant finding of this study is that SAMP8 groups with and without ischemic insult exhibited down-regulation of the VEGF/PDGF signaling system in the brain and that the downregulation could be reversed by CTS administration. VEGF is a hypoxia-inducible secreted protein that interacts with receptor tyrosine kinases such as VEGFR2 on endothelial cells, thereby promoting angiogenesis. Aging is known to cause impairment of angiogenesis *via *an alteration in extracellular matrix and a reduction of angiogenic growth factors such as VEGF [7,9]. In the central nervous system (CNS), VEGF and VEGFR2 are widely expressed not only in vascular endothelial cells but also in neurons, astrocytes, and neural progenitor cells [40]. Moreover, VEGF exerts pleiotropic effects on brain functions including enhancement of adult neurogenesis through the direct activation of neural progenitor cells [42] and ameliorates cognitive deficits *via *the promotion of neurogenesis and its action as a protective factor for endothelial cells and neurons during brain ischemia in adult rats [42,43]. In fact, retardation of angiogenesis in the brain appears to become severe enough in aged animals to impair the neuroplasticity processes since neuroplasticity requires long-lasting increases in metabolic demand supported by the generation of new capillaries [10]. In addition, a recent study by Kim *et al*. [44] revealed that exogenously applied VEGF elevates intracellular Ca^2+^, activates CaMKII and potentiates long-term potentiation in the hippocampal neurons. In this study, we found that the expressions of VEGF and VEGFR2 and their genes were decreased in the brains of older SAMP8, indicating aging-induced dysfunction of the VEGF-VEGFR2 signaling system. Therefore, the impaired VEGF-VEGFR2 signaling systems likely induce a decrease in angiogenesis and neuronal signaling in the brain and are implicated in aging-induced cognitive deficits in SAMP8. Considering a role of the VEGF-VEGFR2 signaling systems in cognitive function, we, in this study, raise the possibility that the CTS-induced reversal of the impaired VEGF-VEGFR2 signaling system is also a part of the mechanism(s) underlying the ameliorative effects of CTS on spatial and non-spatial cognitive deficits caused by aging.

It is of interest to note from the present neurochemical and immunohistochemical studies that the PDGF/PDGFRα system as well as the VEGF/VEGFR2 system was also down-regulated in the brain of the sham- and T2VO-SAMP8 groups compared with that in the control group SAMR1 and that the down-regulation was reversed by the CTS administration in both SAMP8 groups. These findings raise the possibility that aging reduces the function of the PDGF/PDGFR signaling system and that recovery of this system may play a role in the CTS-induced amelioration of cognitive deficits caused by aging with and without ischemic insult. This possibility is supported by a couple of factors. Firstly, evidence indicates that PDGF-A and -B and their receptors (PDGFRα and PDGFRβ) expressed in the CNS [45] are implicated not only in the proliferation, migration and differentiation of oligodendrocytes [46] but also in neurite outgrowth [47], positive and negative modulations of NMDA-receptor function *via *the PKC and PKA pathways respectively [48] and neuroprotection *via *phosphatidylinositol 3-kinase, a mitogen-activated kinase pathway [28]. These signaling mechanisms are important in the long-term potentiation of learning and memory, a biological index of memory formation [49]. Recently, it was reported that PDGFRα and PDGFβ are implicated in the conversion of oligodendroglia progenitor cells to pyramidal neurons in adult piriform cortex [50] and induction of neuroplasticity [51] respectively. Secondly, expression levels of the PDGFs and PDGF receptors and the activation of mitogen-activated protein kinases *via *a PDGF/PDGFR signaling pathway are down-regulated by aging [26]. Taken together with the proposed roles of the PDGF/PDGFR systems in the brain, it is plausible that the CTS-induced reversal of expression levels of PDGF and PDGFR in SAMP8 with and without ischemic insult contributes to the improvement of cognitive performance by CTS administration.

## Conclusion

CTS can ameliorate emotional abnormality and cognitive deficits caused by aging factors including ischemic insults and the recovery of an impaired neuroplasticity system and VEGF/PDGF systems plays an important role in the ameliorative effects of CTS on cognitive dysfunction. CTS is a potential therapeutic agent for aging-related cognitive dysfunctions.

## Abbreviations

CTS: Chotosan; SAMP8: senescence-accelerated prone mice 8; SAMR1: senescence-resistant inbred strain mice; T2VO: transient two vessel occlusion; NMDAR1: N-methyl-D-aspartate receptor 1; CaMKII: Ca^2+^/calmodulin-dependent protein kinase II; CREB: cyclic AMP responsive element binding protein; BDNF: brain-derived neurotrophic factor; VEGF: vascular endothelial growth factor; VEGFR2: VEGF receptor type 2; PDGF-A: platelet-derived growth factor-A; PDGFRα: PDGF receptor α; AD: Alzheimer disease; 3D-HPLC: three dimensional high performance liquid chromatography; ORT: object recognition test; OLT: object location test; PCR: polymerase chain reaction; SDS-PAGE: sodium dodecyl sulfate polyacrylamide gel; GAPDH: glyceraldehyde-3-phosphate dehydrogenase; TBS: Tris-buffered saline; ANOVA: analysis of variance.

## Competing interests

The authors declare that they have no competing interests.

## Authors' contributions

KM designed the study and wrote the manuscript. QZ conducted the behavioral and neurochemical studies. TY participated in the design of the study using SAMP8 and SAMR1. KOT designed the immunohistochemical study and helped analyze the data. KET conducted chemical profiling of the extract. TM conceived the study and helped draft the manuscript. NS participated in the study design and helped draft the manuscript. All authors read and approved the final version of the manuscript.
